# Reduced Surgical Site Infection Rates Following Spine Surgery Using an Enhanced Prophylaxis Protocol

**DOI:** 10.7759/cureus.1139

**Published:** 2017-04-06

**Authors:** Alexa M Dessy, Frank J Yuk, Akbar Y Maniya, James G Connolly, John T Nathanson, Jonathan J Rasouli, Tanvir F Choudhri

**Affiliations:** 1 Icahn School of Medicine at Mount Sinai, Mount Sinai Medical Center; 2 Mount Sinai Medical Center

**Keywords:** surgical site infection, spine surgery, infection, antibiotic prophylaxis

## Abstract

**Background:**

Postoperative surgical site infection (SSI) is a common complication after spine surgery. Reduction of SSI has many benefits including, but not limited to, the reduced length of stay, readmission rates, and morbidity and mortality.

**Objective:**

To determine whether an enhanced antibiotic prophylaxis reduced the rate of surgical site infections in spine surgery.

**Methods:**

This is a retrospective observation study which analyzed the incidence of postoperative SSI following a consecutive series of 1,486 cervical, thoracic and lumbar spine operations performed at a single institution by the senior author between the dates of October 2001 to March 2014. Patients with surgeries between October 2001 and November 2005 received a standard institutional antibiotic prophylaxis. Patients between December 2005 and March 2014 underwent an enhanced antibiotic protocol.

**Results:**

A total of nine cases met the criteria for SSI. All nine cases were recorded during the initial time period when the standard institutional prophylaxis was used. Further, these cases were only observed under posterior operative approaches. No further cases of SSI were observed after the institution of the enhanced antibiotic prophylaxis (p < 0.0001). This was statistically significant in the cervical and lumbar regions (p < 0.0042 and p < 0.0119, respectively).

**Conclusions:**

Although difficult to predict the incidence of SSI, this study found that the use of an enhanced antibiotic prophylaxis protocol significantly reduced one surgeon’s overall rates of surgical site infections after spine surgery.

## Introduction

Postoperative surgical site infection (SSI) is a relatively common complication after spine surgery. The incidence of postoperative infection after spinal surgery has been reported between 0.1% and to 6.7%. Reported rates of SSI vary among different patient populations, procedures, surgeons, and surgical approaches [1-8]. SSI can result in increased morbidity and mortality, the length of hospital stay, increased readmission rate and hospital costs, and post-operative pain. It can also result in and the requirement for an additional surgical procedure, including wound debridement and replacement of hardware [9-13].

Each incidence of SSI can increase the cost of care up to four times the cost of the initial spine surgery [14-17]. Costs are reported to range between $15,800 and $43,900 per SSI [1, 18]. Therefore, efforts to reduce SSI are paramount. In addition, conflicting reports have been published regarding the ideal timing of surgical antimicrobial prophylaxis and the most effective preoperative skin antisepsis [19]. As such, this retrospective review evaluates the incidence of postoperative SSI following spine surgery performed by a single neurosurgeon at a single institution before and after implementation of an enhanced modified prophylaxis protocol.

## Materials and methods

This retrospective study analyzed the incidence of postoperative SSI following a consecutive series of 1,486 cervical, thoracic, and lumbar spine operations performed at a single institution by the senior author in two patient populations that received either the standard prophylaxis protocol or the enhanced prophylaxis protocol between October 2001 and March 2014. From October 2001 until November 2005, patients undergoing spine surgery received the standard institutional protocol. From December 2005 until March 2014, patients undergoing spine surgery received the enhanced protocol (Table 1). Surgical site infections were recorded and defined as per standard of the centers for disease control and prevention (CDC) definitions [20] (Table 2). Inclusion criteria were adult patients undergoing elective cervical, thoracic, or lumbar surgical operations (either primary or revision) by the senior author and the stated date restrictions. Exclusion criteria consisted of only those patients with preexisting infections. Patients were not excluded on the basis of medical comorbidities (diabetes, congestive heart failure (CHF), low serum protein, etc.) or procedure length.

**Table 1:** Standard and enhanced prophylaxis protocol measures Comparison of the standard protocol versus enhanced protocol. Dosage and administration of antibiotics were calculated based on patient’s weight and renal clearance. Standard doses were not used. 1. Two isopropyl alcohol wipes swabbed over incision site and immediate surrounding regions six times. 2. Betadine ointment applied to incision site and immediate surrounding region after use of alcohol wipes. Only used in skin preparation, not after closure. 3. Drains were removed when output reached < 30 cc/eight hour shift.​ 4. Drains used regularly for instrumented cases and selectively for non-instrumented cases.

Indication	Standard Protocol	Enhanced Protocol
Patient Skin Preparation	Standard betadine/iodine scrub/paint. Select use of alcohol pads	Three betadine scrub brushes Six alcohol wipes on incision area^1^ Betadine ointment application^2^
Alcohol Pad Preparation	Select use	Regular use
Patient Prep Performance	Select attending performance	Regular attending performance
Pulse Irrigator (Saline with Bacitracin)	Select use	Regular use for posterior instrumentation
Surgical Drains^3^	Select use	Regular use^4^
Prophylactic Antibiotic Coverage (unless allergic)	IV Cefuroxime for 24 hours Select use of IV Vancomycin (in case of cephalosporin allergy only)	Regular use for the posterior instrumentation: IV Cefuroxime for 24 hoursIV Vancomycin until drain removal Non-instrumentation cases 1. IV Cefuroxime for 24 hours

**Table 2:** Centers for Disease Control and Prevention (CDC) Surgical site infection (SSI) definitions Definition of superficial and deep incisional SSI criteria based on Centers for Disease Control and Prevention [20].

Indication	Superficial Incisional SSI Criteria	Deep Incisional SSI Criteria
Timeline	MUST occur within 30 days after operative procedure	MUST occur within: 30 days of operative procedure if NO implant left in place One year if implant left in place
Tissue Involvement	MUST involve only skin and subcutaneous tissue of the incision	MUST involve deep soft tissues (fascial and muscle layers) of the incision
Drainage, Culture, Symptoms	At least one of the following: Purulent drainage from incision Organisms isolated from aseptically obtained culture At least one of the following signs or symptoms of infection and the superficial incision Pain Redness Tenderness Localized swelling Heat	At least one of the following: Purulent drainage from the deep incision but not the organ space component Deep incision spontaneously dehisces or is deliberately opened by the surgeon and is culture positive OR not cultured when the patient has at least one of the following: Fever Localized pain Tenderness 3) An abscess or other evidence of infection involving the deep incision found on direct examination, during reoperation, or by histopathologic or radiologic exam
Diagnosis	By surgeon or attending physician	By surgeon or attending physician

Data collection consisted of a retrospective review of a patient database of all spinal procedures performed by the senior author drawn from medical records, anesthesia reports, and operative reports. Demographic data included age, sex, body mass index (BMI), American Society of Anesthesiologists (ASA) physical status and length of surgery. These characteristics were chosen for general comparison of the patients under the standard or enhanced protocols and were all measured and recorded from medical and anesthesia records. Cervical, thoracic, and lumbar distinctions as well surgical approach were all defined by the operative location on the patient’s spine. The primary outcome variable evaluated was the incidence of surgical site infection, which was defined as per CDC standards and confirmed with wound cultures. All data were collected and analyzed by independent reviewers uninvolved in the surgical treatment of the patient's study. Statistical analysis involved two-tailed Fisher’s exact tests. Significance was defined using a p-value of less than 0.05. The institutional review board granted approval for this study with waiver of patient consent.

## Results

Over this time frame, a total of 1486 patients met our criteria for inclusion. Table 3 reflects that the patients under the enhanced protocol tend to be older, female and have a slightly higher (BMI/ASA) score compared to the standard protocol group. Table 4 reflects the number of spine surgeries by operative region (cervical, thoracic, and lumbar) and the breakdown of these categories under the use of the standard and enhanced protocols. A total of nine cases (n=nine) met the criteria for SSI as indicated by the criteria set by the CDC (Table 2) for an overall 0.61% infection rate across all spinal surgeries. All cases of SSI occurred under the standard protocol. Of the nine total cases, we observed four cervical, two thoracic, and three lumbar cases of SSI during this time frame. No further cases of SSI were observed during the utilization of the enhanced protocol, with a decline in infection rate from 2.28% under the standard protocol to zero percent under the enhanced protocol (p <0.0001). Likewise, the infection rates declined to zero in each operative region under the enhanced protocol, with infection rates under the standard protocol of 2.44%, 3.51% and 1.73% of cervical, thoracic, and lumbar patients, respectively (p=0.0030, p=0.1487, p=0.0192, respectively). Statistical significance was thus established for overall spine surgery as well as for cervical and lumbar spine surgery. The incidence of SSI was significantly reduced with the use of the enhanced protocol.

**Table 3:** Patient demographics Patient demographics of both cohorts demonstrating similar mean age, percent of male patients, mean body mass index, mean American Society of Anesthesiologist physical status classification and length of surgery.

	Standard Protocol (n=394)	Enhanced Protocol (n=1092)
Mean Age (years)	51.2 ± 16.2	56.9 ± 14.4
% Male	57.9	50.6
Mean BMI	26.88 ± 5.11	27.65 ± 5.61
Mean ASA Status	2.42 ± 0.74	2.54 ± 0.68
Surgery Length (hours)	4.17 ± 2.02	4.23 ± 1.86

**Table 4:** Incidence of surgical site infections by operative region of the spine Incidence of surgical site infections by operative region of the spine shows that the majority of cases were cervical site infections. After the institution of the enhanced protocol, no further surgical site infections were observed.

	Standard Protocol		Enhanced Protocol		Total	
Operative Region	Cases (% Total)	Infections (%)	Cases (% Total)	Infections (%)	Cases (% Total)	Infections (%)
Cervical	164 (41.6)	4 (2.44)	532 (48.7)	0	696 (46.8)	4 (0.57)
Thoracic	57 (14.5)	2 (3.51)	90 (8.3)	0	147 (9.9)	2 (1.36)
Lumbar	173 (43.9)	3 (1.73)	470 (43.0)	0	643 (43.4)	3 (0.47)
Total	394 (100)	9 (2.28)	1092 (100)	0	1486 (100)	9 (0.61)

In addition, all SSI in this study occurred under posterior operative approaches under the standard protocol (Table 5). Table 5 presents the total number of spine surgeries performed under posterior approaches (n=924) and the number of posterior-approach spine surgeries by operative region (cervical, thoracic, and lumbar). Two hundred and thirty-nine posterior surgeries were performed under the standard protocol, and 685 posterior surgeries were performed under the enhanced protocol. The total infection rate with a posterior approach declined from 3.77% to zero percent (p <0.0001). As above, the infection rates of cervical, thoracic, and lumbar surgeries performed under a posterior approach all declined to zero from 5.63%, 4.44%, and 2.44%, respectively (p=0.0042, p=0.1510, p=0.0119, respectively). Cervical, lumbar and total surgeries performed under posterior approaches all reached statistical significance. Thus, the enhanced protocol greatly reduced the incidence of SSI as compared to that under the standard protocol.

**Table 5:** Incidence of surgical site infections by operative region on a posterior approach All cases of surgical site infections were observed using a posterior operative approach. No further cases of SSI were observed after the institution of the enhanced protocol (p < 0.0001) across all regions of the spine. However, statistical significance was reached only for cervical and lumbar cases (p < 0.0042 and p < 0.0119, respectively).

	Standard Protocol		Enhanced Protocol		Total		
Operative Region	Cases (% Total)	Infections (%)	Cases (% Total)	Infections (%)	Cases (% Total)	Infections (%)	P-value
Cervical	71 (29.7)	4 (5.63)	203 (29.6)	0	274 (29.7)	4 (1.46)	0.0042
Thoracic	45 (18.8)	2 (4.44)	70 (10.2)	0	115 (12.4)	2 (1.74)	0.1510
Lumbar	123 (51.5)	3 (2.44)	412 (60.2)	0	535 (57.9)	3 (0.56)	0.0119
Total	239 (100)	9 (3.77)	685 (100)	0	924 (100)	9 (0.97)	< 0.0001

Table 6 displays the microbiology of the surgical wound cultures of all nine cases of SSI, as required by the CDC guidelines. Six of the nine patients and each of the three lumbar and two thoracic patients - with SSI grew methicillin-resistant Staphylococcus aureus from their surgical wound sites. A Staphylococcus species was present in each surgical wound culture with varying degrees of antibiotic susceptibility and coagulase production.

**Table 6:** Surgical wound culture microbiology Six of the nine surgical site infections contained methicillin-resistant Staphylococcus aureus.

Case	Operative Region	Organisms Detected
1	Cervical	Methicillin-resistant *Staphylococcus aureus*
2	Cervical	Methicillin-susceptible *Staphylococcus aureus*
3	Cervical	Coagulase-negative *Staphylococcus* *Escherichia coli*
4	Cervical	Coagulase-negative *Staphylococcus* *Pseudomonas aeruginosa* *Staphylococcus epidermidis*
5	Thoracic	Methicillin-resistant *Staphylococcus aureus* *Staphylococcus epidermidis*
6	Thoracic	Methicillin-resistant *Staphylococcus aureus*
7	Lumbar	Methicillin-resistant *Staphylococcus aureus*
8	Lumbar	Methicillin-resistant *Staphylococcus aureus*
9	Lumbar	Methicillin-resistant *Staphylococcus aureus*

## Discussion

In this study, the incidence of SSI was significantly reduced following the implementation of an enhanced anti-infection protocol. This is supported by the reduced infection rate to zero percent under the enhanced protocol until the end of the patient data query (March 2014). Similar reductions in SSI rates have been observed in several other studies in which protocol modifications were employed [21-23]. However, some studies have suggested that the general incidence of SSI has not substantially decreased in recent years, despite the introduction of evidence-based guidelines for SSI prevention such as the Surgical Care Improvement Project (SCIP) [24-25]. Such findings contrast with the present study, and the results stand to support the implementation of, and compliance with, and evidence-based approach to anti-infection protocols.

Not only does this study support the introduction of more advanced prophylaxis protocols, but it also substantiates the established finding that SSI is more common following posterior spine procedures compared to anterior approaches. No case of SSI was observed in anterior approaches, regardless of protocol. All nine SSIs occurred after posterior procedures. The observed reduction in SSI in posterior approaches supports our claim that stringent use of this anti-infection protocol reduced the rate in these surgeries. The study, however, was not powered appropriately to determine which operative region of posterior approaches presented the greatest risk of postoperative infection. This underscores the need to understand the incidence of SSI in the context of the given risk factors for the particular procedure involved.

Identifying patient specific pre-operative risk factors are essential for future research to develop an evidence-based approach to SSI. The multifaceted nature of this changed and enhanced protocol hinders our ability to derive which component contributed most substantially to the reduced infection rate. Evidence suggests that multiple of the enhancements may affect the prevention of SSI, from preoperative skin preparation protocol to irrigation to antibiotic usage [14, 15, 21, 22, 26-28]. Future studies should include analysis by type of surgery and indication for surgery.

In addition, as a retrospective review, the validity of our study rests primarily on the quality and nature of the data collection. The database utilized for this study, however, was produced and analyzed by independent reviewers uninvolved in the surgical treatment of the study’s patients and thus unbiased toward the results.

## Conclusions

An evidence-based understanding of patient-specific and procedure-specific risk factors, as well as specific facets of the protocol itself, remains to be seen. Additional analyses are warranted to quantitatively analyze patient comorbid factors and procedure characteristics to develop an algorithm to predict the relative risk of infection for any given patient. Further investigation is needed to compare the results with patient outcomes in other practices within the neurosurgery department and with other institutions. With this in mind, we recommend future study and appropriate use of this enhanced protocol.
